# Contributions of information and communications technology to future health systems and Universal Health Coverage: application of Japan’s experiences

**DOI:** 10.1186/s12961-020-00585-x

**Published:** 2020-06-26

**Authors:** Shuhei Nomura, Vera Siesjö, Göran Tomson, Wiebke Mohr, Eriko Fukuchi, Kenji Shibuya, Viroj Tangcharoensathien, Hiroaki Miyata

**Affiliations:** 1 Department of Global Health Policy, Graduate School of Medicine, The University of Tokyo, Tokyo, Japan; 2 Department of Health Policy and Management, School of Medicine, Keio University, Tokyo, Japan; 3 Institute of Global Health Policy Research (iGHP), National Center for Global Health and Medicine, Tokyo, Japan; 4 Swedish Institute for Global Health Transformation (SIGHT), Royal Swedish Academy of Sciences, Stockholm, Sweden; 5 ACCESS Health International, Manila, Philippines; 6 Department of Learning, Informatics, Management and Ethics (LIME), Karolinska Institutet, Stockholm, Sweden; 7 Department of Healthcare Quality Assessment, Graduate School of Medicine, The University of Tokyo, Tokyo, Japan; 8 International Health Policy Program, Ministry of Public Health, Bangkok, Thailand

**Keywords:** Future health system, Universal health coverage, Information and communications technology, PeOPLe

## Abstract

**Background:**

Demographic changes in the pattern of disease burden, escalating health expenditures and inequitable access to healthcare are global challenges. Irrespective of their level of development, all countries need to reform their health systems to prepare for the future emerging health needs, in order to meet their commitments of health systems strengthening, universal health coverage (UHC) and explicit targets in the Sustainable Development Goals (SDGs).

**Summary:**

We propose three core principles for the future health system as described herein. A health system is not simply a ‘cure delivery machine’ but part of a ‘social security system’ that engages all stakeholders through a shared vision and value of health and well-being, not merely an absence of diseases. The future health system shall provide people-centred, affordable care, tailored to the individual’s needs, accessible at any time and any place, and reflect the notion of leaving no one behind through a life course approach — underpinned by the SDGs. Information and communications technology (ICT) offers the potential to facilitate the realisation of these principles by improving the information flow between different parts of the health system through electronic means. We introduce Japan’s new data platform — Person-centred Open PLatform for wellbeing (PeOPLe) — planned to be introduced in 2020 as one example of an ICT-based intervention to realise the three proposed principles. PeOPLe integrates data collected throughout the life course to enable all people to receive affordable, personalised health and social care at any time and any place throughout their lifetime. Furthermore, we discuss the applicability of these principles and PeOPLe to the health systems context of Thailand and the Philippines, including elaborations on ICT transformation challenges.

**Conclusion:**

Current rising momentum and scale for ICTs in the UHC era offers a great opportunity to make a difference for countries. The PeOPLe concept is not only relevant to resource-rich countries; its applicability to other Asian countries could be feasible though it will need to be adapted to the various country contexts. We hope that this paper contributes to wider discussion around policy choices of ICT application for future health systems strengthening and UHC in order to achieve the SDGs.

## Background

Despite a remarkable improvement in global public health over the past decades [1], challenges emerge from demographic and epidemiologic transitions dominated by non-communicable diseases (NCDs) along with the escalating health expenditures and increased inequities in access to healthcare [1]. This stands out particularly in low- and middle-income countries (LMICs), which are facing a triple burden of disease due to underachieved Millennium Development Goals, targets of maternal and child mortality reduction, and persisting high rates of communicable diseases as well as emerging NCDs, all of these while facing fiscal constraints [2].

**Fig. 1:**
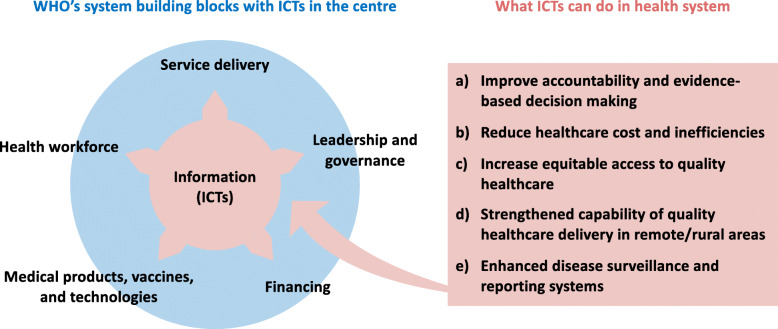
Using ICTs to accelerate health system strengthening. To best conceptualise the ICTs as a foundation for the ‘health information’ building block in the WHO’s health system framework [4], the framework was modified to have ICTs in the centre with all other building blocks around it. The five key functions that ICTs could contribute in this context were based on the recent literatures [5, 6]

Irrespective of their level of development, all countries need to reform their health systems to prepare for the future emerging health needs and keep up with their commitment to health systems strengthening (HSS), Universal Health Coverage (UHC) and the Sustainable Development Goals (SDGs) [3]. WHO defines HSS as “*improving and combining six key health system strengthening building blocks in ways that achieve more equitable and sustained improvements across health services and outcomes*” (Fig.1) [7]. UHC is defined as a system where all people have equitable access to quality health services without financial hardship [8], which aligns with SDG Target 3.8.1 (equitable access to health) and Target 3.8.2 (financial risk protection). UHC is a major driver for achieving other health-related SDGs such as poverty reduction (SDG 1), nutrition improvement (SDG 2) and gender equality (SDG 5) [9]. There is no single approach to UHC; different countries have different strategies. In light of the recent 40th anniversary of 1978 Alma Ata Declaration, the commitment to the Astana Declaration on primary healthcare presents an unparalleled opportunity for action towards UHC through primary healthcare strengthening. However, recent evidence shows that achieving health-related SDG targets requires a concerted shift from disease-centred curative interventions towards more multisectoral, prevention-oriented policy action that addresses the social determinants of health, while honouring the promise of leaving no-one behind [10, 11].

**Fig. 2:**
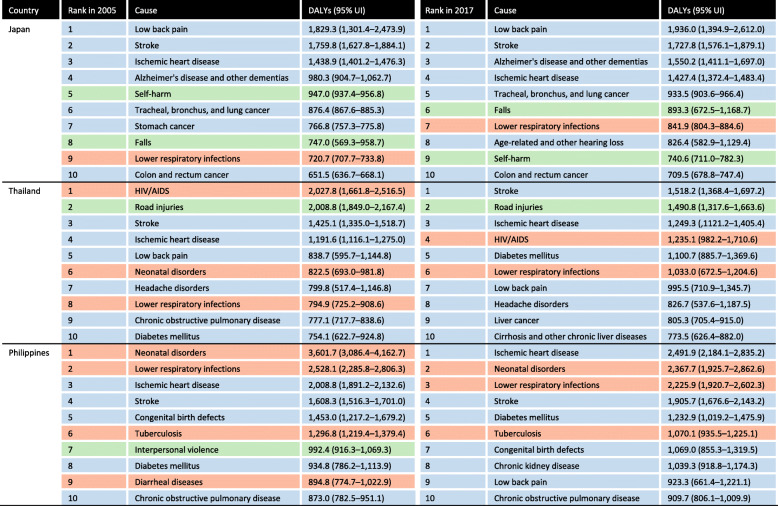
GBD level 3 causes of DALYs per 100,000 population in Japan, Thailand and the Philippines in 2005 and 2017. Source: GBD 2017 [1]. *DALYs* disability-adjusted life-years; *GBD* Global Burden of Disease, Injuries, and Risk Factors, *UI* uncertainty interval. Orange: communicable, maternal, neonatal, and nutritional diseases; Blue: non-communicable diseases; Green: injuries. Ranking is based on the DALYs per 100,000 population

Japan achieved UHC by 1961. Today, Japan shows excellent health outcomes at relative low health spending (current health expenditure of 10.9% of gross domestic product (GDP) in 2016 compared to the average among Organisation for Economic Co-operation and Development countries at 12.6% of GDP [12]) while maintaining a high level of health equity [13]. Despite these achievements, Japan faces serious fiscal constraints primarily due to stagnant economic growth, combined with an increasing older population, rising health expenditures, and a decrease in the number of young people with the ability to bear taxes due to the population decline (Supplementary file 1) [14]. In fact, as the population ages, the disease burden of disabling conditions (e.g. low back pain, pain from cervical spondylosis and sensory disorders) and degenerative diseases (especially Alzheimer’s disease) is increasing rapidly (Fig. 2) [1], putting a strain on the health system, especially in terms of financing [15]. In 2015, a Japanese government advisory panel of young experts proposed the 2035 vision of the Japanese health system — Japan Vision: Health Care 2035 [16]. The panel proposed paradigm shifts from a disease-centred towards a people-centred care model and encouraged all relevant stakeholders to apply and maximise the use of information and communications technology (ICT) to support future health systems. WHO defines people-centred care as “*an approach to care that consciously adopts the perspectives of individuals, families, and communities, and sees them as participants as well as beneficiaries of trusted health systems that respond to their needs and preferences in humanistic and holistic ways*” [17]. In June 2018, the Japanese Cabinet adopted the ‘Future Investment Strategy 2018’, which promotes a reform towards the next generation health system based primarily on ICT-based interventions [18].

Through reviews and syntheses of the literature, this paper first discusses the potential contributions of ICTs to HSS as one of the core pillars to support UHC. Second, we propose three principles for the future health systems and how ICTs can facilitate the realisation of these principles, using Japan’s new data platform — Person-centered Open PLatform for wellbeing (PeOPLe) — as an example of an ICT-based intervention. Third, we discuss the relevance of these principles and the applicability of PeOPLe to the context of the Philippines and Thailand. The purpose of this paper is to contribute to the discussion on policy options of ICT application to HSS and, consequently, the achievement of UHC.

## Potential of ICT for HSS in the UHC era

ICT is defined as “*a family of technologies used to process, store and disseminate information, facilitating the performance of information-related human activities, provided by and serving both the public at-large as well as the institutional and business sectors*” [19]. Examples of ICT applications include the Internet of Things, Artificial Intelligence, cloud computing, big data, consumer mobile applications, etc. An ICT-based intervention is defined in this paper as a discrete functionality of ICT and is implemented within ICT applications.

‘Information’, an essential part of ICT, is one of the six health system building blocks that drive effective health systems, as suggested by WHO (Fig. 1) [4]. Robust health systems are the foundations for moving towards UHC and other health-related SDGs. Access to high-quality and reliable information enable the efficient functioning of the other five building blocks. It supports identification of problems and needs, making evidence-based policy decisions, monitoring and evaluation of health programmes, and equitable allocation of scarce resources.

Integrating ICTs into health systems can improve the flow of information between different parts of health systems and sectors beyond health [20]. According to recent findings [5, 6], ICTs contribute to improved access, service coverage transparency, reduced healthcare costs, improved efficiency and enhanced social security (Fig. 1). Hence, ICT could be viewed as a backbone of the three dimensions of UHC (population covered, services covered and costs covered), all of which require effective approaches to data and information management to optimise the use of limited resources. As outlined in the recently published WHO guideline on ICT-based interventions for HSS [21], strategic harnessing of ICTs is essential to make rapid and affordable progress towards HSS and UHC as well as for the achievement of the SDGs. Yet, barriers to ICT integration, such as the lack of infrastructures, affordability, political support, and human capital in LMICs, need to be addressed to maximise the potential of ICT for HSS and UHC [22].

## Future health system direction

Health, influenced by a complex interplay of various determinants of health and wellbeing in a society [23], is a precondition for and an outcome and indicator of all three dimensions of sustainable development — economic, social and environmental. It is not merely a matter of healthcare, but a critical means of comprehensive human development. Japan’s ‘Health Care 2035’ vision aims to realise a society that empowers people to make the healthcare choices that are best for them while providing responsive care through diverse social networks, including family, community, school and workplace, i.e. supporting people to play an active role in maintaining their own health [16]. Still, caution is necessary as health systems are highly context dependent (see below). We propose three core principles for the future health system, to ultimately achieve a society in line with ‘Health Care 2035’. In Table 1, we conceptualise and propose the three principles for a future health system based on the four Ws (what, where, who and when), comparing past and present.

**Table 1:** Four W questions addressing the past and present and proposing the future of health systems

4 Ws	Past	Present	Future
Where	Hospital-based health system	Community-based health system	Anywhere in a social system
What	Acute care	Long-term care	People-centred care (personalised, affordable, accessible)
When	In the case of acute and communicable diseases	In the case of chronic and non-communicable diseases	At any time
Who	Ambulatory patients (younger population)	Elderly patients (older population)	All people, leaving no one behind, through a life course approach

### Principle 1 – Health system as a core part of a social system

A health system should be guided by the broader context of the social determinants of health, including the principles of biomedical ethics, fairness, equity and human rights. It has to be a core part of a ‘social security system’ that connects actors and engages stakeholders beyond the health sector through a shared vision and value of health and well-being throughout the life course [16].

### Principle 2 – People-centred care, anytime, anywhere

The future health system should be more people centred, tailored towards the individual’s emerging needs, including social support. Health systems should be affordable and accessible at any time and any place. People centred refers to ensuring that the health system is responsive to and respectful of the individual. We also believe it includes the empowerment of people and communities to be competent in the use of ICTs.

### Principle 3 – All people, leaving no one behind, through a life course approach

The future health system should adhere to the notion of ‘leaving no one behind’ throughout the complete life course. A governance that encompasses all health system actors and the people of all ages who are the users of services contributes to a people-centred health system [24]. Based on the above, including the three principles, and guided by the SDGs, we now introduce one example of ICT-implementation for HSS in UHC.

## PeOPLe – ICT-based intervention for the future health system in Japan

The ‘PeOPLe’ data platform is Japan’s new state-of-the-art ICT-based intervention that aims to contribute to HSS and UHC by making the best use of health data and tackle health systems constraints in the country (Supplementary file 1). This initiative was proposed in October 2016 and was led by a government advisory panel (including authors of this article) for promotion, application and integration of ICTs into health systems; it is expected to start operation from 2020 [25]. PeOPLe refers to an open data digital health platform on the internet that integrates various existing electronic health and healthcare data collected throughout the life course. Users, such as patients and healthcare professionals, can log in to the online digital platform using internet-enabled devices (smartphones, tablets, computers, etc.). On the platform, users can access individuals’ data in strict confidentiality according to the laws and regulations applicable in PeOPLe (described below). The information will include basic information on disease, medication and therapy history (e.g. insurance claim data) as well as pre-diagnostic data (e.g. periodic health check-up data and life logs, such as sleep time, drinking, food intake, etc.). A unique individual citizen identifier will play a critical role in PeOPLe by linking all different data platforms through an internet-connected device, to identify, analyse and make the best use of information to benefit the individual.

The PeOPLe ICT-based platform may play a significant role in boosting the function of the WHO’s ‘Information’ building block (Fig. 1). Recognising the three principles for a future health system and leveraging the benefits of information as one of the health system building blocks (e.g. cost reduction, operational efficiency and improvement of processes within the health system, etc., as described above), PeOPLe aims to make all individuals have access not only to personalised, affordable healthcare but also to social care, which addresses social determinants of health beyond disease management, at any time and any place throughout their lifetime.

PeOPLe aims to apply an open application programming interface, which allows PeOPLe to seamlessly integrate many healthcare and social applications and enables all stakeholders, including researchers, healthcare professionals, various industries and companies, authorities, service providers, patients, and decision-makers, to use the accumulated big data (in anonymised form) for public health research and policy purposes (Supplementary file 2) [26]. The application programming interface system could improve equitable access to health and social care. The system aims to ensure cost reduction through the efficient use of resources and easier and faster on-demand deployment of care services. PeOPLe can be powered by artificial intelligence systems to aid users in decision-making, streamlining business processes and operations, optimising resource use, and reducing administrative complexity and costs [27]. Further information on PeOPLe design, challenges and solutions can be found in Supplementary file 3.

## Addressing challenges of the application of ICT for HSS

While ICT-based interventions in health could be key enablers to make rapid progress towards HSS, UHC and the achievement of several SDGs, there are cautions that should be considered when applying ICT-based interventions to future HSS.

### Context dependency of health systems

Vision for future health systems and application of ICTs should take the healthcare and social systems context into account [28]. Health and social systems are shaped by national socioeconomic and political, historical, and cultural structures. Hence, the application of ICT is not only about making technology accessible and inclusive, but it requires a social consensus on the purpose of a future health system and the application of ICT to support HSS.

### Transformational challenges of ICTs

ICT transformation is about the adoption of ICTs in society at large. The way in which society realises the transformational potential of ICTs is not free from obstacles. The diffusion of useful ICT-based interventions is supported by inexpensive ways to access ICTs and their application (e.g. mobile technologies), the use of computerised decision-making systems and algorithms, increased integration of public and private ICT-based services, or other regulatory issues [29, 30].

### Use of ICTs by people with mental and physical limitations

People with mental and physical limitations can benefit hugely from ICTs in enhancing their autonomy in society [31]. However, it is well recognised that the need for assistive technologies, including ICTs, is high but demand is low. A lack of supply to match demands by these populations is a concern, especially in LMICs due to inadequate budget allocation to support access to these technologies [32]. Government leadership, financial commitment and multisectoral approaches can overcome these challenges [32].

Older persons, frailties, persons with disabilities and those who are less ICT literate could benefit from PeOPLe, but sufficient assistance in training of usage of these technologies needs to be provided to ensure equitable access.

### Security, trust and privacy concerns around ICTs

Data protection is an integral part of people-centred care as it is a fundamental right of all people to have their personal data protected. Data privacy must be carefully considered, especially in LMICs, where privacy and data protection rules are often not yet established or enforceable in the social security system [33].

During the last years, several legal regulations have been endorsed to protect the privacy of individuals. For example, the General Data Protection Regulation (GDPR) by the European Union (EU), effective since of May 2018, strengthens citizens’ fundamental rights and facilitates business by simplifying rules for one single digital market. According to the European Commission, businesses in the EU, until recently, dealt with 28 different data protection laws, which represented a large, costly administrative burden [34]. The Commission estimated that simplification of the regulatory environment through the introduction of the GDPR would save approximately 2.3 billion euros per year in costs for business [35].

Data protection in Japan is governed by the Act on the Protection of Personal Information, which was enacted in 2003. Japan amended the Act in 2015 as part of the privacy reform and it has been now recognised as ‘equivalent’ to the GDPR in terms of the level of protection of personal data [36]. PeOPLe will incorporate these laws by specifying the privilege of data use according to the nature of various users’ objectives in different parts of the health system to access the platform. Adhering to several provisions of the Act on the Protection of Personal Information, all information collected on PeOPLe from individuals will be protected and personal identification information will be removed prior to being extracted out of the platform, providing an anonymised dataset to the user. Individuals will have a right to control over how their data is used (e.g. what information is distributed to whom and for what purposes and for which use it is permitted) and will have the right to rectify erroneous or inaccurate data. No data containing personal identification information will be publicly available. This will make the platform secure and all information collected will be kept strictly confidential to the extent permitted by the applicable laws and regulations. Thus, everyone, whether it is an individual, company, government, healthcare provider or insurer, can securely use the platform.

## Applicability of PeOPLe to the health systems context in Thailand and the Philippines

### Thailand

Thailand is globally recognised for its successful UHC. As a result of the Universal Coverage Scheme introduced in 2002, the current population coverage has reached 99.9% [37]. However, as in many other countries with similar socioeconomic conditions, control of infectious diseases has been successful and NCDs now represent the major causes of disease burden (Fig. 2) [1]. Meanwhile, the rate of ageing in the population in the country is outpacing that of Japan and health expenditures are increasing (from 3.2% of GDP in 2000 to 3.8% in 2015) [38]. Hence, the government recognises the needs for a sustainable future health system [39].

Our proposed principle 1 of the ‘health system as a social system’ is being applied in Thailand, especially for the management of older patients with NCDs [40]. For example, similar to North America, Europe and some high-income countries in Asia [10], Thailand is making efforts to promote multisectoral approaches that integrate healthcare into social care frameworks in order to maintain healthy aging and well-being among its population in response to the rise in NCDs, addressing the commercial determinants of NCDs [41]. This includes daily life support as well as provision of end-of-life care in community care settings and home visits by family care teams for those who cannot access healthcare services. Even though access to assistive technologies was adequately in line with the implementation of the UN Convention on the Rights of Persons with Disabilities [42, 43], the benefits of such integrated care models are limited to older and disabled people, still inaccessible in rural and hard to reach communities [40]. Adequacy and financial sustainability are required for a more inclusive society [40].

Through Thailand’s three public health insurance schemes, the country has a fully developed national inpatient dataset of all discharges, approximately 7 million inpatients a year, using a 13-digit Citizen Identification Number (CID) — a lifetime unique identifier given by the registration office at birth. The national inpatient dataset is also used for reimbursement under a diagnostic related group system by all three insurance schemes. Hence, there exist large datasets on risk behaviours, clinical diagnoses, laboratory test results, use of medicines and surgical interventions, and clinical outcomes such as mortality and disability. Other locally initiated disease registries, such as renal replacement therapy, tuberculosis and antiretroviral therapy, also apply a CID, where health professionals can provide seamless continued services through security-protected access to these registries from anywhere in the country at any time, using the internet platform (Supplementary file 2). This facilitates people-centred services in line with principle 2. The application of tele-medicine for clinical diagnoses such as radiology, histology and consultations from remote areas without specialists, and for access support to orphan medicines such as antidotes for life-threatening conditions [44], are examples of ICT applications ensuring equitable access to all by leaving no one behind, in line with principle 3.

Thailand currently works together with Japanese partners under a Memorandum of Understanding to design a roadmap for developing an ICT-based data platform (similar to PeOPLe). The platform aims to create a people-centred, affordable health system in the country by integrating various electronic healthcare data, as described above, linked with the CID [45].

In February 2019, the first Thailand Personal Data Protection Act was approved and endorsed by the National Legislative Council, which has modified several concepts from the EU’s GDPR [46]. Thailand has, to date, established 957 secured internet servers per million population, which is much lower than Japan (11,682 servers per million people) [47]. Its extensive work in the health sector in order to implement an ICT like PeOPLe is likely to further facilitate the establishment of secured servers. Although PeOPLe is just one example of an ICT-based intervention, Thailand shows high applicability of an ICT like PeOPLe and relevance of the proposed principles despite being a developing country.

### Philippines

As the Philippines continues to battle infectious diseases, it faces a triple burden of disease, with an increasing disease burden of NCDs as well as a persisting high burden of child mortality (Fig. 2) [1]. The signing of the Universal Health Care bill in February 2019 was seen as a major milestone in the move towards provision of coverage and access to healthcare services for all Filipinos. The signing of the UHC bill is much in line with our proposed principle 1 of ‘health system as a social system’, by integrating health and social services. The bill aims to automatically enrol all Filipinos into the national health insurance programme of the Philippine Health Insurance Corporation [48]. The Philippine Health Insurance Corporation is currently tasked with working on major health system challenges in the country, namely high, unaffordable out-of-pocket expenditures and supply-side constraints as well as inequitable and limited access to quality care [49]. In order to tackle these challenges, the government recognises the need to maximise resources to provide a sustainable future health system.

In the Philippine Health Agenda 2016–2022, ICT interventions are one of the main pillars and, as reflected in principle 1, the Agendas 2016–2022 address all life stages and the triple burden of disease by implementing a multisectoral approach beyond health that addresses the broader array of social determinants of health [50]. In accordance with the agendas, in January 2019, a bill to establish the National eHealth Systems and Services was passed to deliver health services through cost-effective and secure ICTs (Supplementary file 2) [51].

The Philippines is at an initial phase of piloting a unique national identifier. In August 2018, the Philippine Identification System Act was signed, which formalises the implementation of a Philippine Identification System (PhilSys) [52]. Similar to PeOPLe, PhilSys will provide all citizens as well as resident aliens with a lifetime unique identification number, authenticating the citizens’ identities in all government and private sector transactions [53]. However, compared to PeOPLe, the proposed system lacks integrated functions that allow the patients to access personal medical and health data. PhilSys seeks to unify all government IDs into one, and healthcare and social care services are aimed to be tied in with the use of the national PhilSys ID. The aim of PhilSys is aligned with our principle 3 by providing faster access to services for all and leaving no one behind.

Meanwhile, the National Privacy Commission ensured that the protection of the data privacy rights is a top priority in the implementation of the PhilSys. If the Philippines are to implement a similar system to PeOPLe, the current Data Privacy Act of 2012 is not adequate to protect citizens’ privacy as evidenced by the country’s history of data breaches [54]. Although the Philippines has made progress on the application of ICTs to achieve UHC and a more people-centred healthcare system, the applicability of PeOPLe and the relevance of our proposed principles of the future health system will depend on future progress of UHC and the rigors of personal privacy data laws.

## Conclusions

Today, sustainable access to quality, equitable and affordable health services is a major challenge. Health systems around the globe require a significant paradigm shift away from the disease-centred service focus in order to achieve UHC based on the three proposed core principles, namely that (1) a health system is not simply a ‘cure delivery machine’ but part of a ‘social security system’ that engages all stakeholders through a shared vision and value of health and well-being, not merely an absence of diseases. Further, the future health system shall provide (2) more people-centred, affordable care, tailored to the individual’s needs, accessible at any time and any place, and reflect the notion of (3) leaving no one behind throughout the life course — underpinned by the SDGs.

ICT, when integrated into HSS and UHC, can serve as a facilitator for the realisation of these principals by improving the information flow between different parts of health systems and sectors beyond health, through electronic means. Japan’s PeOPLe is one example of ICT-based interventions and can serve as inspiration when rethinking the way of how to reform countries’ health systems to prepare for the future emerging health needs along with the power of information that can be accessed by individuals. Several caveats need to be considered before the ICT-based interventions are operational in health systems and become fully utilised, especially in poor resource settings — context dependency of health systems, transformational challenges of ICTs, limited access to ICTs by the people with mental and physical limitations, and security and privacy concerns around ICTs. Current rising momentum and scale for ICTs in the UHC era offers a great opportunity to make a difference for countries. As described by the Thailand and Philippines cases, the PeOPLe concept may not only be relevant to resource-rich countries. We hope that this paper contributes to a wider discussion around policy choices of ICT application to future HSS and UHC to achieve the Agenda 2030 for sustainable development.

## Supplementary information

**Additional file 1: Supplementary file 1.** Japan’s health system challenges in the SDG era. **Supplementary file 2.** Implications of ICT on business, research, and policy. **Supplementary file 3.** Design, challenges and solutions for PeOPLe.

## Data Availability

Not applicable.

## Abbreviations

CID: Citizen Identification Number EU European Union GDP Gross domestic product GDPR General Data Protection Regulation HSS Health system strengthening ICT Information and communications technology LMICs Low- and middle-income countries NCD Non-communicable disease PeOPLe Person-centered Open PLatform for wellbeing PhilSys Philippine Identification System SDGs Sustainable Development Goals UHC Universal health coverage
