# Supplementary material for: Contributions of information and communications technology to future health systems and Universal Health Coverage: application of Japan’s experiences
Source: Health Res Policy Syst. 2020 Jun 26;18:73. doi: 10.1186/s12961-020-00585-x (PMC7318469; doi:10.1186/s12961-020-00585-x)
Supplement: Supplementary file 1 — Additional file 1: Supplementary file 1. Japan’s health system challenges in the SDG era. Supplementary file 2. Implications of ICT on business, research, and policy. Supplementary file 3. Design, challenges and solutions for PeOPLe. [file 12961_2020_585_MOESM1_ESM.docx]

**Supplementary 1: Japan’s health system challenges in the SDG era**

Japan's exemplary health accomplishment in the past 50 years has been characterized by universal insurance scheme, where people are free to choose health care facilities, and good quality of care at comparably low cost.^[1]^ While equity is emphasized as the societal core value and health policy, health gain is achieved by addressing social determinants of health.^[1]^ However, health financing is hampered by economic stagnation, which has also widened socio-economic disparities.^[2]^ As inequalities increase, social determinants of health will likely deteriorate, leading to poorer population health and greater demand for health care services.^[3]^ Combined with rising health care expenditure (from 7.2% of the gross domestic product in 2000 to 10.9% in 2015^[4]^) and an aging population, it is crucial for Japan to ensure the sustainability of equitable, quality, and affordable health care access under such pressures. Under these circumstances, reconfiguring the health system only for a cost reduction will end up affecting its fair accessibility and the quality of the health care.^[5]^

Major challenges in the Japan health system also include a large reliance on professional ethos in health care quality and efficiency rather than on evidence-based, data-driven governance, and decision-making.^[6]^ Japan's policy of tight control of health care cost under a nationally uniform fee schedule and a market-based (rather than service needs) approach to service delivery, likely create a mismatch between needs and supply of health care services, which are the least cost-effective and far from being people-centred, and impede governance and accountability for quality of services.^[6]^ This system was formed based on rapid economic growth after World War II.

With the slowdown in economic growth and the coming unprecedented aging society, Japan's health care policies urgently need a systematic evaluation to identify and correct bottlenecks in the today's health system at the personal, institutional, and system-wide levels. Despite the growing appetite for mature information infrastructure (including big data) for health system research and evidence-based decision-making and policy formulations, fragmentation of data flow occurs because of silos of data collection, analysis, interpretation, and dissemination of outcome data.

**Supplementary 2: Implications of ICT on business, research, and policy**

In Japan, medical institutions, insurers, local governments, companies, and others have their own data on patients and citizens' healthcare and other life-logs using different systems. This distribution of data among stakeholders impedes effective data utilization. However, by sharing data among them through the PeOPLe platform, they will be able to work together, for example, to establish new business models, accelerate measures to improve the well-being of local communities, develop robust scientific research, and critically evaluate policy decisions.

In Thailand, applications of ICT for disease registries such as tuberculosis, end-stage renal failure on renal replacement therapy, HIV on anti-retroviral treatment contribute policy and clinical practice for improve effective coverage,^[7]^ through identification and mitigation of the barriers. These registries also contribute to extensive clinical research for improve clinical outcomes and increase five-year survival. The introduction of an ICT like PeOPLe will allow the integration of the databases for these disease registries, which will enable a wide range of research, policy, and business use of the registries. For example, the discovery of symptoms of diseases and the research and commercialization of methods to prevent them will lead to further improvement of the population health in the country.

In Philippine, in January 2019, a bill to establish the National eHealth Systems and Services was passed to deliver health services through cost-effective and secure ICTs.^[8]^ In The National eHealth System and Services Act, a consolidation of six measures that are aligned with the WHO building blocks and the achievement of UHC, similarly to PeOPLe, aims to integrate the information flow of health care data. It could potentially be used in the longer term in the development of effective preventative healthcare business, research and policy measures. However, it currently lacks the infrastructure and investment needed to function optimally. The system is similar to PeOPLe in its intents to facilitate the access to and exchange of personal health information, and ensure integration, alignment, and interoperability among various eHealth initiatives. Equally to PeOPLe, it aims to provide more transparency and equitable access to services, throughout the life course, by linking all citizens to an insurance.^[8]^

In the future health system where our three principals have been realized through the benefits of ICTs like PeOPLe, patients and individuals will be more actively involved in maintaining and improving their health and will design them by themselves. Accordingly, health policy plans and their outcomes will need to be widely shared and continually evaluated not only by governments and service providers but also by all stakeholders, including patients, private sector, academic institutions, etc. In addition, in order to maximize the benefits of ICTs, policy makers (of central and local governments) need to make necessary legal and regulation reforms (e.g. for the data protection through data sharing and consolidation on PeOPLe), develop human resources, and strengthen administrative functions. They

**Supplementary 3: Design, challenges and solutions for PeOPLe**

Balancing data protection and public interest

PeOPLe is based on the concept that individuals should be able to distribute and manage their own data at their own will. At the same time, PeOPLe is promoting the distribution and use of data held by governments, companies and other organizations by linking them to individuals.

Individual data are sensitive and processing needs to take adequate steps to protect individual rights and interests, which are under consideration.^[9]^ For example, participation in PeOPLe will be based on the consent of each citizen, and a sufficient security environment will be prepared, such as setting a difference in the degree of anonymization processing of data and requiring human and physical safety management measures in the data usage environment, depending on the purpose and form of utilization at the data provider. However, the need for flexible operation is also being considered for the use of anonymously processed information with opt-out consent and for purposes that are in the public interest (e.g., during disasters).

At the same time, from the viewpoint of transparency in data utilization, for example, a mechanism is needed in which individuals can see who has accessed their information (e.g. access logs). Furthermore, from the viewpoint of the right to control personal information, it is necessary to set the scope of which personal information should be shared with whom. It is important to balance the utilization of the data and the protection of private information of the nation.

Research shows that Japan has a higher level of public anxiety about providing personal data than the US, UK and Germany.^[10]^ In order to promote PeOPLe, it will be important to promote public education on the use of PeOPLe and propose individual convenience. In addition, the intention to use related services and applications that utilize individuals’ data is low and acceptable level of providing individuals’ data (i.e. under what conditions) is also low.^[10]^ Establishing a system that allows people to enjoy beneficial services by providing data aims to lead to the creation of a virtuous cycle of data utilization. In order for the information to be properly viewed and managed by the individual, there is a need to understand the value of the data and the consequences of providing the data. In addition, it is necessary to make the public aware that health-related data is not only useful for the benefit of the individual, but also has the benefit of being a "public good". The data can be useful for the development of medicine, social medicine, public health strategies, health policy, etc. In order to create such an environment, it is important that not only the government, but also various companies, academia, and medical professionals work together.

Data standardization

In the case of an individual-centered data distribution platform like PeOPLe, data standardization is important. For example, electronic medical records provided by different vendors have a wide variety of systems and formats, and each health care provider has different management codes and entry methods, making "data standardization" which facilitates “data inter-operability” a major issue when collecting, sharing, and using data. In order to promote the integration of data in PeOPLe, it is necessary to promote the standardization of basic information. Although yet not tested for its functionality, PeOPLe aims to share information between systems efficiently by using an open application programming interface (API).

Bibliographies

1. The Lancet. Japan: universal health care at 50 years. *The Lancet* 2011; **378**(9796): 1049.

2. Shibuya K, Hashimoto H, Ikegami N, et al. Future of Japan's system of good health at low cost with equity: beyond universal coverage. *The Lancet* 2011; **378**(9798): 1265-73.

3. World Health Organization. Closing the gap in a generation: health equity through action on the Social Determinants of Health : Commission on Social Determinants of Health final report: World Health Organization, Commission on Social Determinants of Health; 2008.

4. Organisation for Economic Co-operation and Development. Health at a Glance 2017 [Internet]. 2017 (accessed 2020 June 01). <https://www.oecd-ilibrary.org/social-issues-migration-health/health-at-a-glance-2017_health_glance-2017-en>.

5. Reich MR, Shibuya K. The Future of Japan's Health System--Sustaining Good Health with Equity at Low Cost. *The New England Journal of Medicine* 2015; **373**(19): 1793-7.

6. Hashimoto H, Ikegami N, Shibuya K, et al. Cost containment and quality of care in Japan: is there a trade-off? *The Lancet* 2011; **378**(9797): 1174-82.

7. Ng M, Fullman N, Dieleman JL, Flaxman AD, Murray CJ, Lim SS. Effective coverage: a metric for monitoring Universal Health Coverage. *PLoS Med* 2014; **11**(9): e1001730.

8. Umali T. House panel approves proposed “National eHealth System and Services Act” [Internet]. 2019 (accessed 2020 June 01). <https://www.opengovasia.com/house-panel-approves-proposed-national-ehealth-system-and-services-act/>.

9. Ministry of Health, Labour and Welfare. Points to consider when considering PHR from the perspective of citizens and patients [Internet]. 2020 (accessed 2020 June 01). <https://www.mhlw.go.jp/stf/newpage_09355.html>.

10. Ministry of Internal Affairs and Communications. 2017 White Paper on Information and Communications in Japan. Tokyo: Ministry of Internal Affairs and Communications, 2017.
